# Supplementary material for: The relative role of direct orbital forcing versus CO2 and ice feedbacks on Quaternary climate
Source: Nat Commun. 2026 Mar 19;17:4254. doi: 10.1038/s41467-026-70750-3 (PMC13168665; doi:10.1038/s41467-026-70750-3)
Supplement: Supplementary file 1 — Supplementary Information [file 41467_2026_70750_MOESM1_ESM.pdf]

The relative role of direct orbital forcing versus CO_2 and ice feedbacks on Quaternary climate

C. J. R. Williams, N. S. Lord, A. T. Kennedy-Asser, X. Ren, D. A. Richards, M. Crucifix, A. Kontula, M. Thorne, P. J. Valdes, G. L. Foster, R. M. Brown, E. L. McClymont and D. J. Lunt.

Supplementary Material

TEXT

(S1) Additional details on testing the emulator: LGM approach

As part of the emulator testing (see Methods, in particular Last Glacial Maximum approach), we compared the LGM SAT anomaly from the emulator with the original LGM HadCM3 simulated SAT anomaly (Figure S3a and b, respectively), and against two LGM SAT anomaly reconstructions[1,2] (Figure S3c and d, respectively). Both of these reconstructions comprise a proxy data assimilation into the climate model iCESM1.2 and 1.3; although the former study calculates LGM anomalies relative to the late Holocene (defined as 0-4 kyr)[1], they state that this can be considered representative of PI conditions.

There is good agreement between the emulated and reconstructed SATs, with the most significant regions of cooling located over the Laurentide ice sheet in North America (Fig. S3). Discrepancies include the extent of cooling over the Laurentide and Fennoscandian ice sheets, which is geographically comparable in size to the former study[1] but not as large as the latter study[2]. HadCM3 at this time has a global mean cooling value of 5.3°C, slightly cooler than the traditional consensus range of 2-4.5°C[1]. In contrast, the latter study found a global mean cooling of ~6-7°C[2]. Therefore, if the former reconstruction[1] is taken to be correct, the emulated SAT projections during glacial conditions could therefore be considered as being somewhat cold-biased; but if the latter reconstruction[2] is taken to be correct, then the emulated SAT projections could instead be considered as being somewhat warm-biased. However, it is worth noting that the temperatures over ice sheets in both reconstructions are uncertain, due to the lack of direct proxies in these regions.

(S2) Additional details on linear factorisation

The linear factorisation method consists of incrementally adding in one driver at each step, to create a single pathway from a no drivers simulation to an all drivers simulation[3]. The drivers are switched on (i.e. using real data, denoted by a 1) or switched off (i.e. a constant value taken at PI levels, denoted by a 0) in the following order: CO_2, obliquity, eccentricity, precession and GSL/ice (see Table S2 for all 32 simulations). For example, Figure S6 shows one such linear factorisation, in which each individual driver was added in turn, to transition from E_{00000} to E_{11111}. This is only one possible linear factorisation, or pathway, among other possibilities; this particular pathway involves adding one driving component incrementally i.e. E_{00000}, E_{10000}, E_{11000}, E_{11100}, E_{11110}, E_{11111}).

This point is made more clearly, and indeed globally, by Figure S7, where a single pathway is considered. Here, it is clear that the impacts of CO_2 (Fig. S7a) and ice (Fig. S7e) are having a much greater contribution to the overall signal (as measured by the M score compared to the all drivers simulation) than any of the individual orbital components, with the latter (Fig. S7b-d) contributing less than half of the overall signal across much of the world. This is consistent with the multi-variate factorisation method is used (see Figure 3 in main manuscript) which, unlike linear factorisation, compares all possible pathways within the factorisation, averaging across each dimension to get to the mean contribution from each driver[3]. In certain places CO_2 and ice are contributing up to all of the overall signal, such as across the tropics for CO_2 (Fig. S7a). The ice component is providing the most contribution (accounting for almost all of the overall signal) over the polar latitudes (Fig. S7e). When compared to the proxy data, it is only when the CO_2 and ice components are added into the emulator does the temperature match the magnitudes suggested by the reconstructions, qualitatively suggesting that ice and CO_2 are providing the majority contribution to the all drivers simulation (Fig. S6).

(S3) Additional details on creating the training data

Global temperatures during the Pliocene were higher than they are at present, meaning that in the ice sheet reconstruction the GrIS is limited to high elevations in the Eastern Greenland Mountains, and no ice is present over Western Antarctica[4]. Large regions of the East Antarctic ice sheet (EAIS) show minimal changes or slightly increased surface elevation, although there is substantial loss of ice in the Wilkes and Aurora subglacial basins[4]. Similar patterns of ice retreat have been simulated in response to future warming scenarios for the GrIS[5] and WAIS[6]. The two ice sheet configurations used here therefore represent climate

states ranging from interglacial conditions to relatively severe global warming conditions accompanied by significant ice sheet retreat.

Other ice sheet extents reconstructions are available[7], as well as results from recent ice sheet models[8], which could result in changes to the simulated climate. For example, some studies have suggested a lower maximum elevation for the Laurentide ice sheet during glacial conditions[9]. Modelling studies have suggested that the topography of the ice sheet can have a significant impact on mean sea level pressure, which affects the location of the wind-driven gyre circulation in the subpolar North Atlantic and on the strength of the AMOC, resulting in warming in the North Atlantic[10]. Storm tracks in the North Atlantic have also been shown to be affected, with associated impacts on precipitation and snowfall in northern Europe[10]. However, we do not expect these uncertainties to impact our main conclusions, in terms of the relative importance of the various drivers.

(S4) Additional HadCM3 details

The atmospheric component of HadCM3 is coupled to the land surface scheme MOSES2.1 (Met Office Surface Exchange Scheme), which is in turn coupled to the dynamic vegetation model TRIFFID (Top-down Representation of Interactive Foliage and Flora Including Dynamics)[11]. TRIFFID calculates the global distribution of vegetation based on competition between five plant functional types: broadleaf trees, needleleaf trees, C3 grasses, C4 grasses and shrubs.

Although no longer considered state-of-the-art, compared to, for example, those GCMs included in IPCC Sixth Assessment Report[12], HadCM3 is fairly computationally efficient which makes it appropriate for running experiments that cover periods of several centuries or longer, as well as for running ensembles with a large number of ensemble members, as is required in this study. For this reason, the model is still widely used in climate research, particularly in palaeoclimate studies[13]. It has also previously been used in conjunction with a statistical emulator to investigate climate sensitivity to palaeoclimate forcing[14].

(S5) Additional details on parameter details and optimisation

As part of the optimization process, the performance of an emulator was assessed by how well it reproduced the HadCM3 SAT data that it was trained on. The input factors ($\ln(CO_2)$, ε, $e\sin\varpi$, $e\cos\varpi$, and GSL) were standardised prior to the calibration being performed; each

was centred in relation to its column mean, and then scaled based on the standard deviation (SD) of the column. Different emulator configurations were tested by varying the number of PCs retained, ranging from 5 to 20, and for each emulator configuration, the correlation length scales δ and nugget ν were optimised by maximisation of the penalized likelihood. This optimisation was carried out in log space, ensuring that the optimised hyperparameters would be positive. After some testing (see below), the two (one interglacial and one glacial) emulator configurations that performed best were selected as the final two optimised emulators. It was found that the optimised interglacial emulator retained 15 PCs (accounting for 90% of the total variance), and had length scales δ of 2.792 (ε), 1.310 ($e\sin\varpi$), 1.664 ($e\cos\varpi$), 0.523 (CO_2), and 10.000 (GSL), and a nugget of 0.000. The optimised glacial emulator also retained 15 PCs (accounting for 81% of the total variance), and had length scales δ of 6.908 (ε), 7.499 ($e\sin\varpi$), 5.460 ($e\cos\varpi$), 1.003 (CO_2), and 0.290 (GSL), and a nugget of 0.050.

TABLES

		Emulated				
		**Dome C**	**ODP 1012**	**ODP 1123**	**ODP 1239**	**ODP 806**
**Proxy**	**Dome C**	620.018	143.853	87.3801	103.833	74.3982
	**ODP 1012**	209.391	327.926	321.399	360.423	273.541
	**ODP 1123**	98.5815	116.177	128.205	137.756	77.904
	**ODP 1239**	86.0269	150.248	197.004	200.483	157.456
	**ODP 806**	29.36	130.769	88.7556	121.533	128.86

Table S1. Arcsin Mielke (M) scores between emulated and proxy data, for E_{11111} (see Table S2), at every possible combination of Ocean Drilling Program (ODP) sites.

	Description
E_{00000}	All drivers held constant at PI levels
E_{00001}	GSL/ice varying, other drivers held constant at PI levels
E_{00010}	Precession varying, other drivers held constant at PI levels
E_{00011}	GSL/ice and precession varying, other drivers held constant at PI levels
E_{00100}	Eccentricity varying, other drivers held constant at PI levels
E_{00101}	GSL/ice and eccentricity varying, other drivers held constant at PI levels
E_{00110}	Precession and eccentricity varying, other drivers held constant at PI levels
E_{00111}	GSL/ice, precession and eccentricity varying, other drivers held constant at PI levels
E_{01000}	Obliquity varying, other drivers held constant at PI levels
E_{01001}	GSL/ice and obliquity varying, other drivers held constant at PI levels
E_{01010}	Precession and obliquity varying, other drivers held constant at PI levels
E_{01011}	GSL/ice, precession and obliquity varying, other drivers held constant at PI levels
E_{01100}	Eccentricity and obliquity varying, other drivers held constant at PI levels
E_{01101}	GSL/ice, eccentricity and obliquity varying, other drivers held constant at PI levels
E_{01110}	Precession, eccentricity and obliquity varying, other drivers held constant at PI levels
E_{01111}	GSL/ice, precession, eccentricity and obliquity varying, other drivers held constant at PI levels
E_{10000}	CO_2 varying, other drivers held constant at PI levels
E_{10001}	GSL/ice and CO_2 varying, other drivers held constant at PI levels
E_{10010}	Precession and CO_2 varying, other drivers held constant at PI levels
E_{10011}	GSL/ice, precession and CO_2 varying, other drivers held constant at PI levels
E_{10100}	Eccentricity and CO_2 varying, other drivers held constant at PI levels
E_{10101}	GSL/ice, eccentricity and CO_2 varying, other drivers held constant at PI levels
E_{10110}	Precession, eccentricity and CO_2 varying, other drivers held constant at PI levels
E_{10111}	GSL/ice, precession, eccentricity and CO_2 varying, other drivers held constant at PI levels
E_{11000}	Obliquity and CO_2 varying, other drivers held constant at PI levels
E_{11001}	GSL/ice, obliquity and CO_2 varying, other drivers held constant at PI levels
E_{11010}	Precession, obliquity and CO_2 varying, other drivers held constant at PI levels
E_{11011}	GSL/ice, precession, obliquity and CO_2 varying, other drivers held constant at PI levels
E_{11100}	Eccentricity, obliquity and CO_2 varying, other drivers held constant at PI levels
E_{11101}	GSL/ice, eccentricity, obliquity and CO_2 varying, other drivers held constant at PI levels
E_{11110}	Precession, eccentricity, obliquity and CO_2 varying, other drivers held constant at PI levels
E_{11111}	All drivers varying

Table S2. Description of which drivers vary and which are held at preindustrial (PI) levels for each of the 32 simulations.

Ensemble	Number of simulations	Input parameters			
		Orbital		Atmospheric CO_2 (ppmv)	Ice sheets
modice	60	ε	22.2 : 24.4	250 : 1901	Modern
		Esinϖ	-0.055 : 0.055		
		Ecosϖ	-0.055 : 0.055		
lowice	60	ε	22.2 : 24.4	250 : 1901	PRISM4 Pliocene
		Esinϖ	-0.055 : 0.055		
		Ecosϖ	-0.055 : 0.055		
highice	62	LGC		LGC	ICE-5G LGC

Table S3. Input parameter set-ups for the modern, low and high ice (modice, lowice and highice, respectively; the latter including the Last Glacial Cycle, LGC) climate model ensembles, including sampling ranges for the orbital parameters of obliquity, precession and eccentricity (ε, Esinϖ and Ecosϖ, respectively), atmospheric carbon dioxide in parts per million (CO_2 ppmv) and ice sheets.

FIGURES

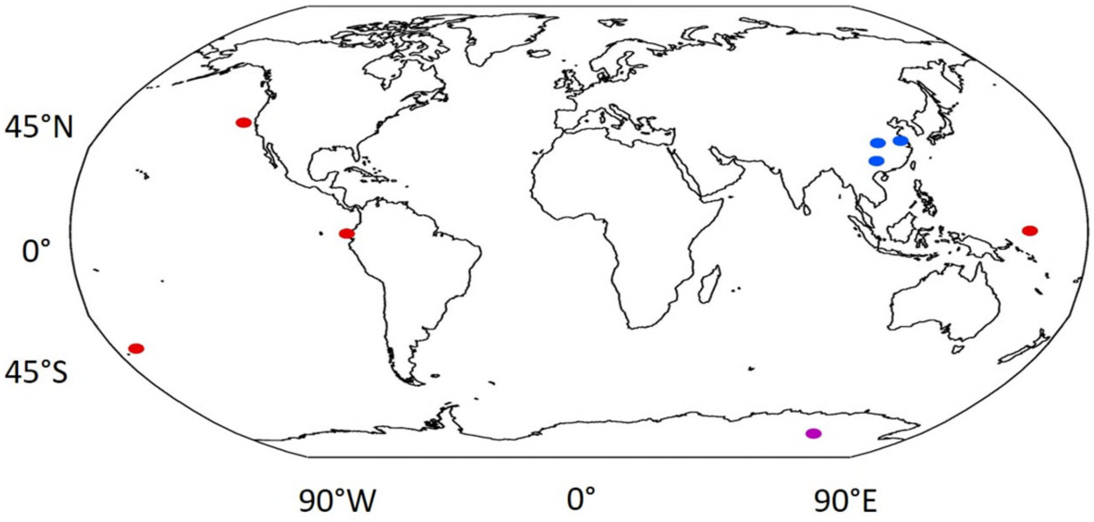

Figure S1: Proxy data locations. Map of the source locations of the proxy data records: purple dot = surface air temperature (SAT)[15,16], blue dots = precipitation[17,18,19], red dots = sea surface temperature (SST)[20,21,22,23], See Table 1 for details.

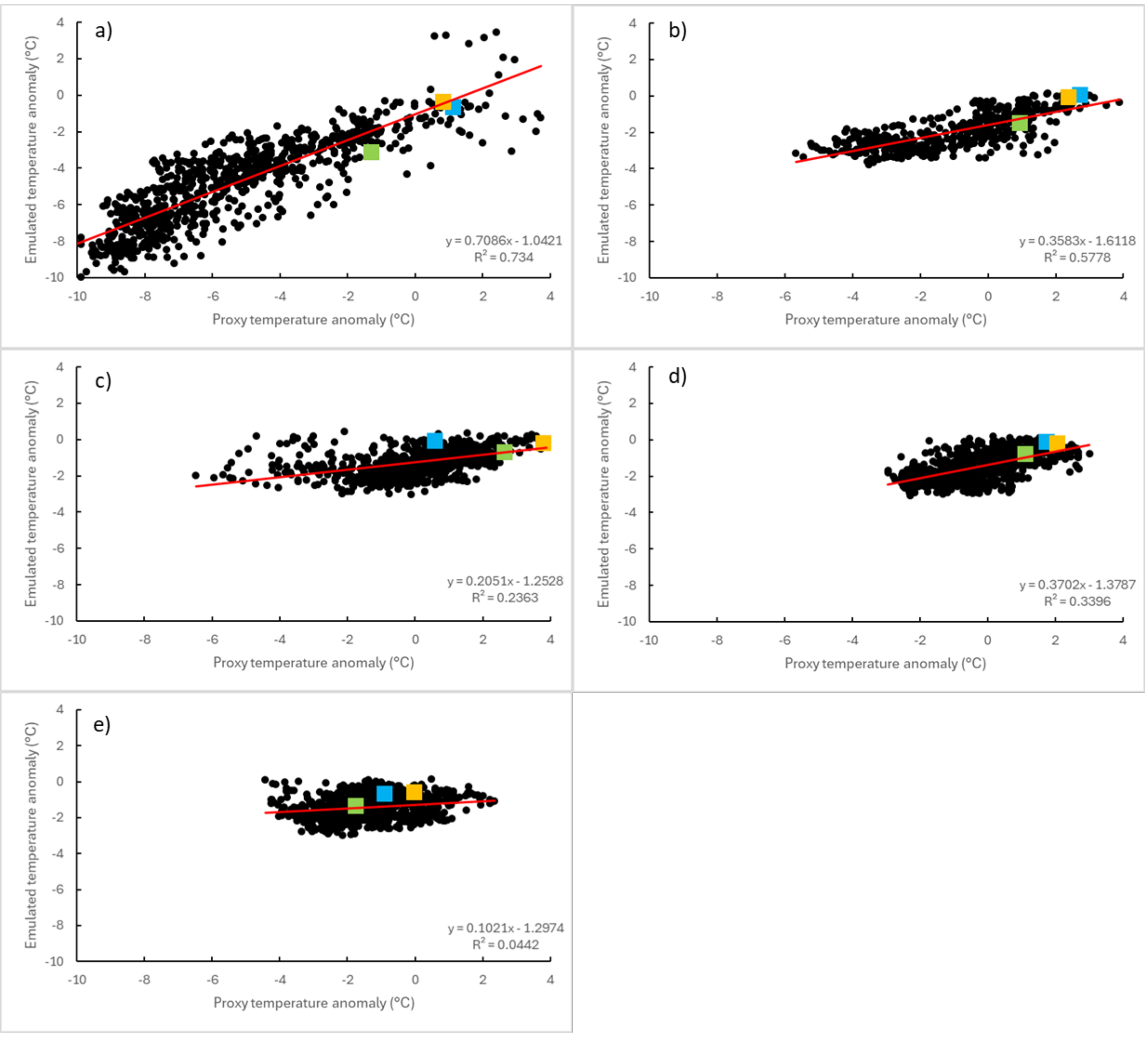

Figure S2: Model-data comparison. Scatterplot of temperature anomalies (compared to a preindustrial control simulation (i.e. 0 thousand years (kyr)), °C) for the last 800 kyr at five locations, reconstructed from proxy data and modelled every 1 kyr using the emulator E_{11111} simulation (see Methods, Section 5.3 for more details**)**: a) surface air temperature (SAT) from the Dome C ice core, Antarctica; b) sea surface temperature (SST) at Ocean Drilling Program (ODP) 1012, north-east Pacific; c) SST at ODP 1123, central South Pacific; d) SST at ODP 1239, Equatorial East Pacific; e) SST at ODP 806, Equatorial West Pacific. See Table 1 for details. All SST shown as an anomaly compared to an average of the first 3 kyr from the dataset. Blue, green and orange larger squares show Marine Isotope Stage (MIS) 5, 7 and 9, respectively.

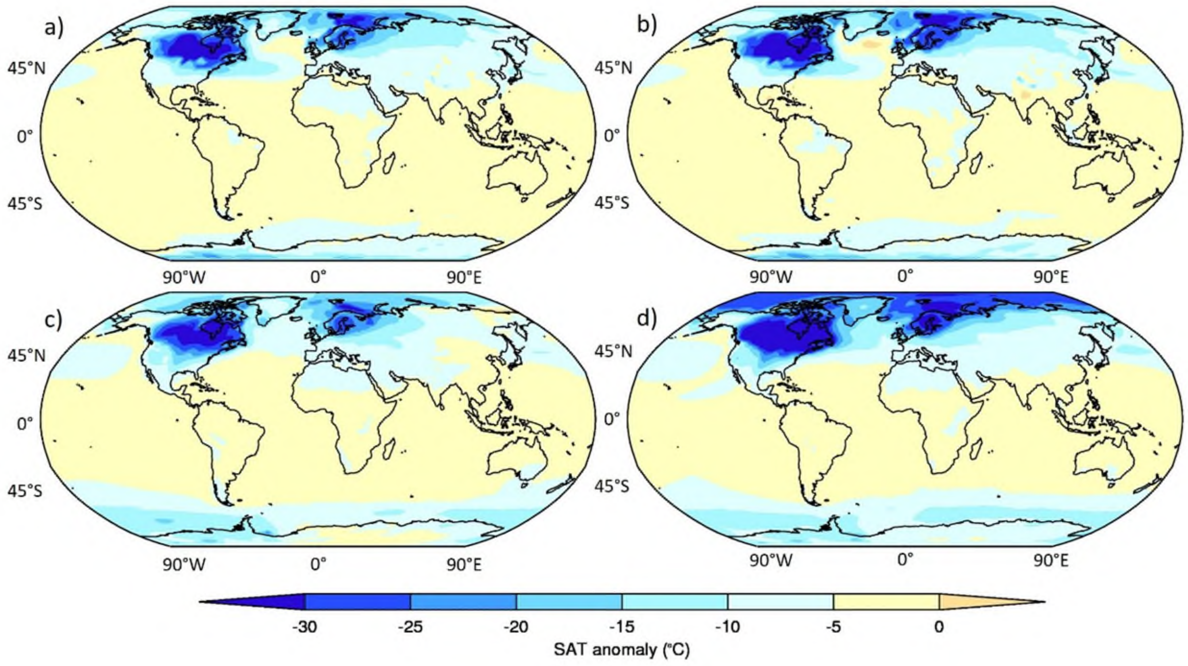

Figure S3: Last Glacial Maximum (LGM) comparison. Maps of surface air temperature (SAT) anomaly (compared to pre-industrial in a, b and d and compared to the late Holocene in c; °C) at the LGM (21 kyr BP) as projected by: a) the emulator 3; b) HadCM3; c) first data assimilation[1] and d) second data assimilation[2], both of which use assimilate proxy data into a climate model.

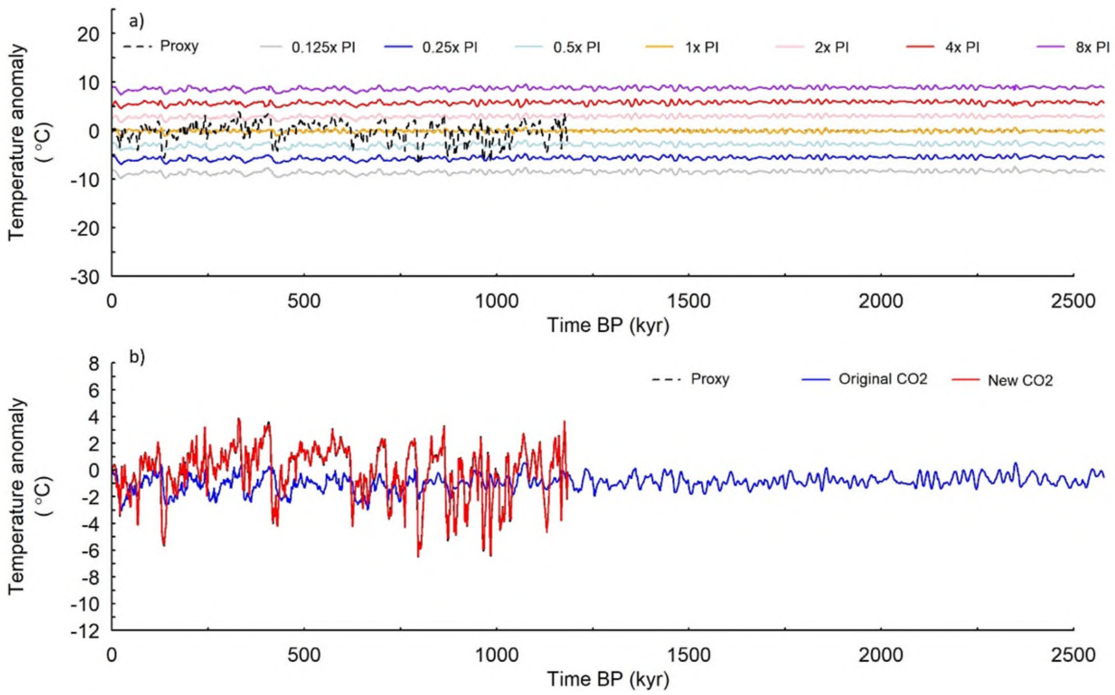

Figure S4: Atmospheric, dioxide (CO_2) generation. Examples from the CO_2 generation process: a) Timeseries of emulated (coloured lines) and reconstructed (dashed line) surface air temperature (SAT) anomalies (°C) for the last 2.58 million years (Myr) in the central South Pacific, using the constant CO_2 inputs from the idealised simulations; b) Timeseries of emulated (coloured lines) and reconstructed (dashed line) SAT anomalies (°C) for the last 2.58 Myr in the central South Pacific, showing the emulated SAT resulting from the original (i.e. reconstructed) CO_2 and the new CO_2. The proxy temperatures are mostly invisible, because the emulated temperatures (as a result of running with the optimised CO_2) are identical.

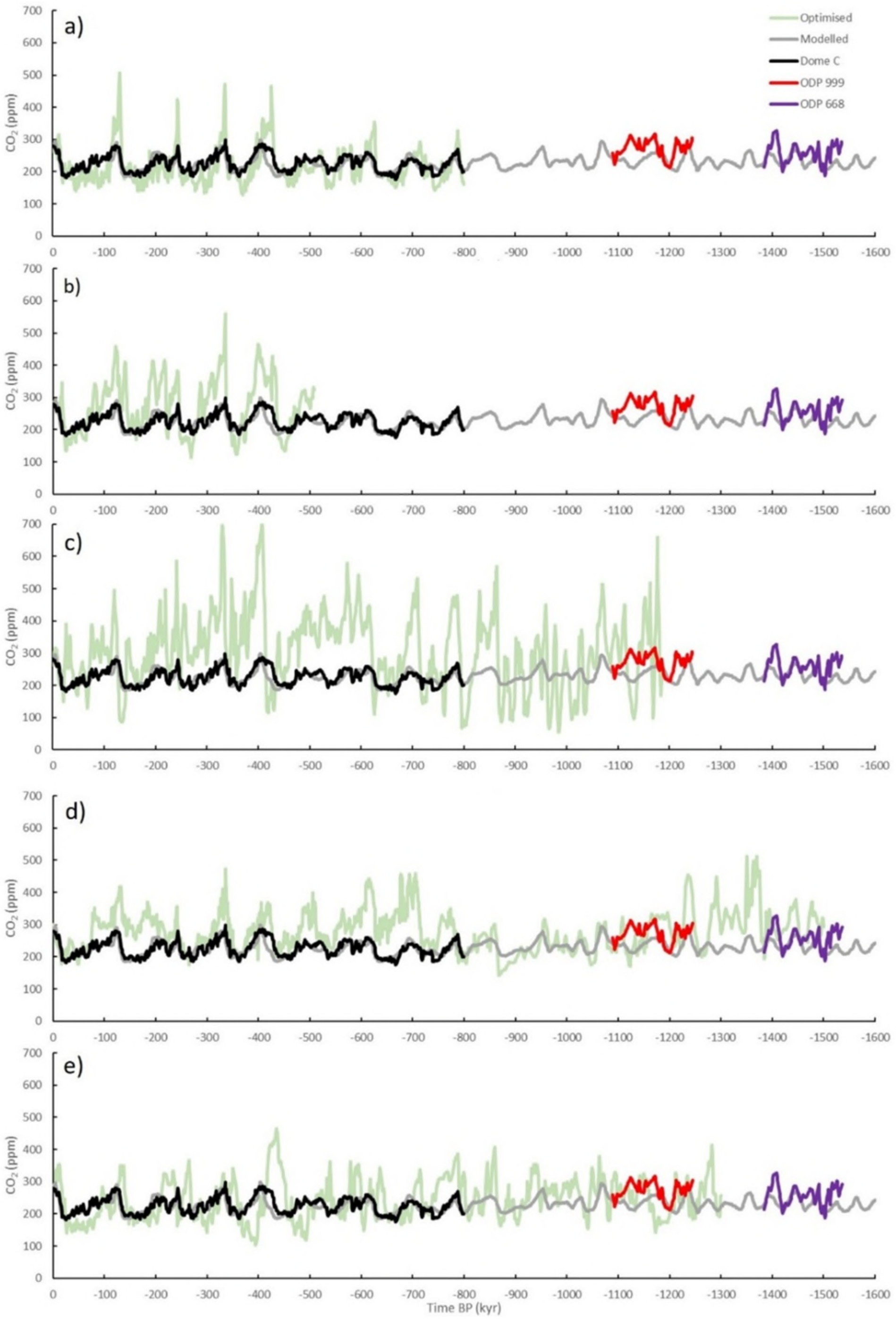

Figure S5: Atmospheric carbon dioxide (CO_2)-data comparison. Timeseries of CO_2 (ppm) for the last 1.6 million years (Myr), showing proxy CO_2 (black, red and purple lines), modelled CO_2 (grey line) and new CO_2 (green line) created by interpolating from proxy temperature reconstructions at five locations: a) surface air temperature (SAT) from the Dome C ice core, Antarctica; b) sea surface temperature (SST) at Ocean Drilling Program (ODP) 1012, north-east Pacific; c) SST at ODP 1123, central South Pacific; d) SST at ODP 1239, Equatorial East Pacific; e) SST at ODP 806, Equatorial West Pacific.

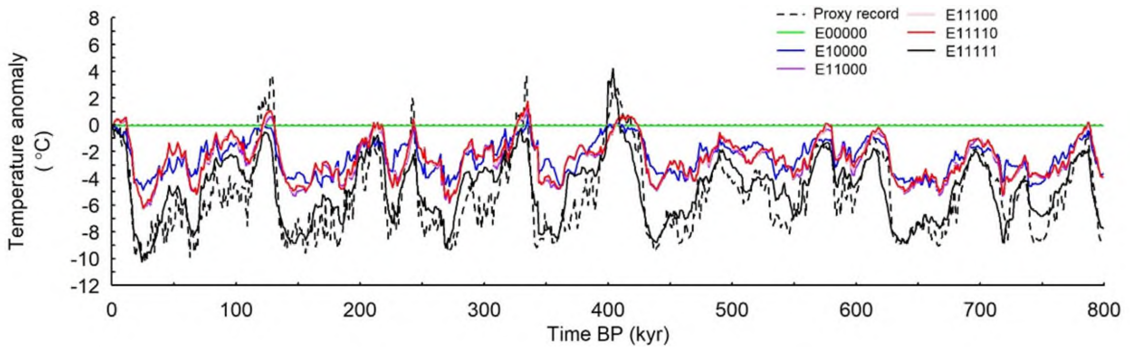

Figure S6: Timeseries of linear factorisation. Timeseries of surface air temperature (SAT) anomaly (°C) for the last 800 thousand years (kyr) at Dome C, Antarctica, reconstructed from proxy data (dashed black lines) and modelled every 1 kyr using the emulator, which was forced by each simulation (solid lines) from the first pathway in the linear factorisation (in which each driving component is added incrementally, see Methods).

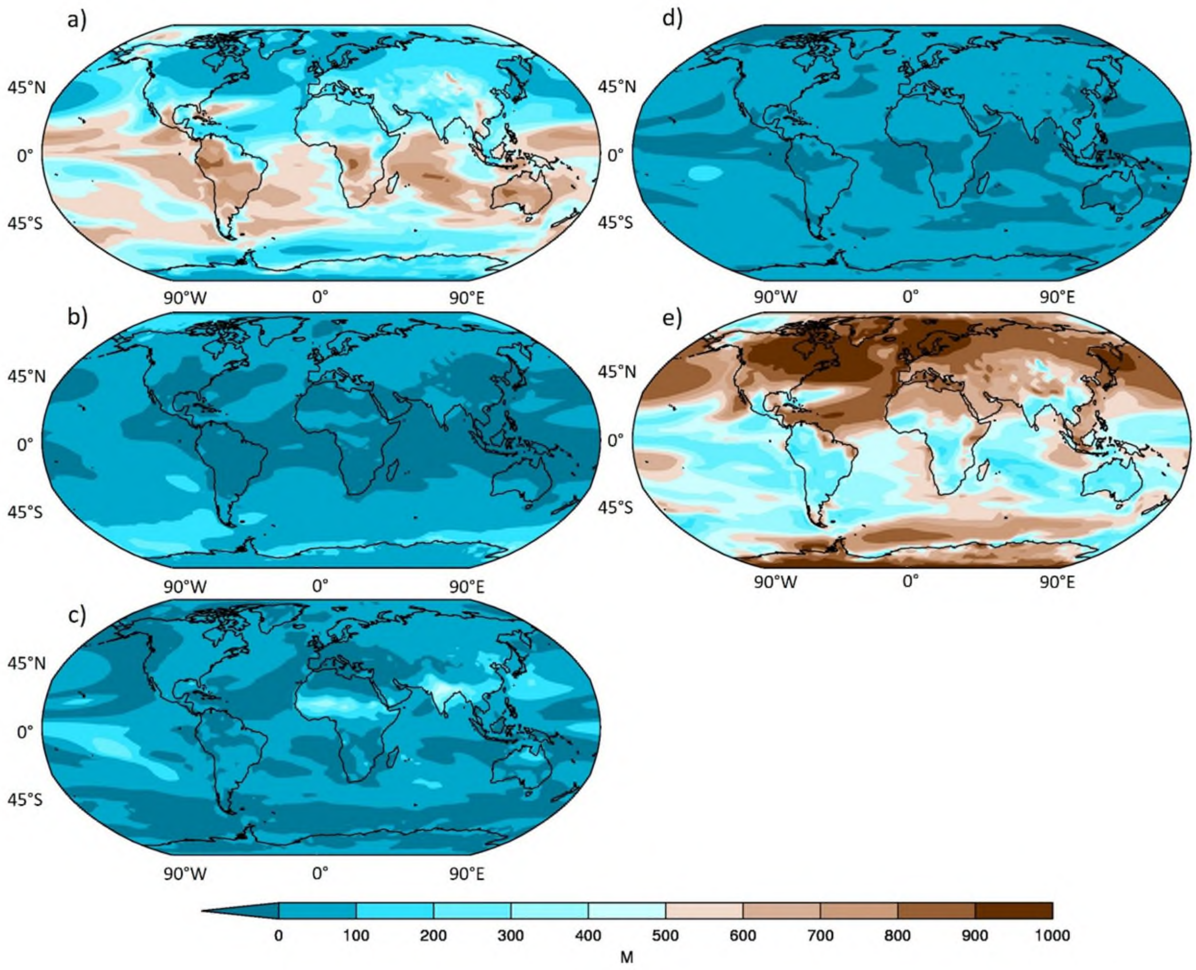

Figure S7: Maps of linear factorisation. Arcsin Mielke (M) scores between emulated temperature from the all drivers simulation (E_{11111}) (see Methods, Section 5.3 for more details) and emulated temperature from the first pathway in the linear factorisation, for each driving component: a) atmospheric carbon dioxide (CO_2); b) Obliquity; c) Eccentricity; d) Precession; e) Ice.

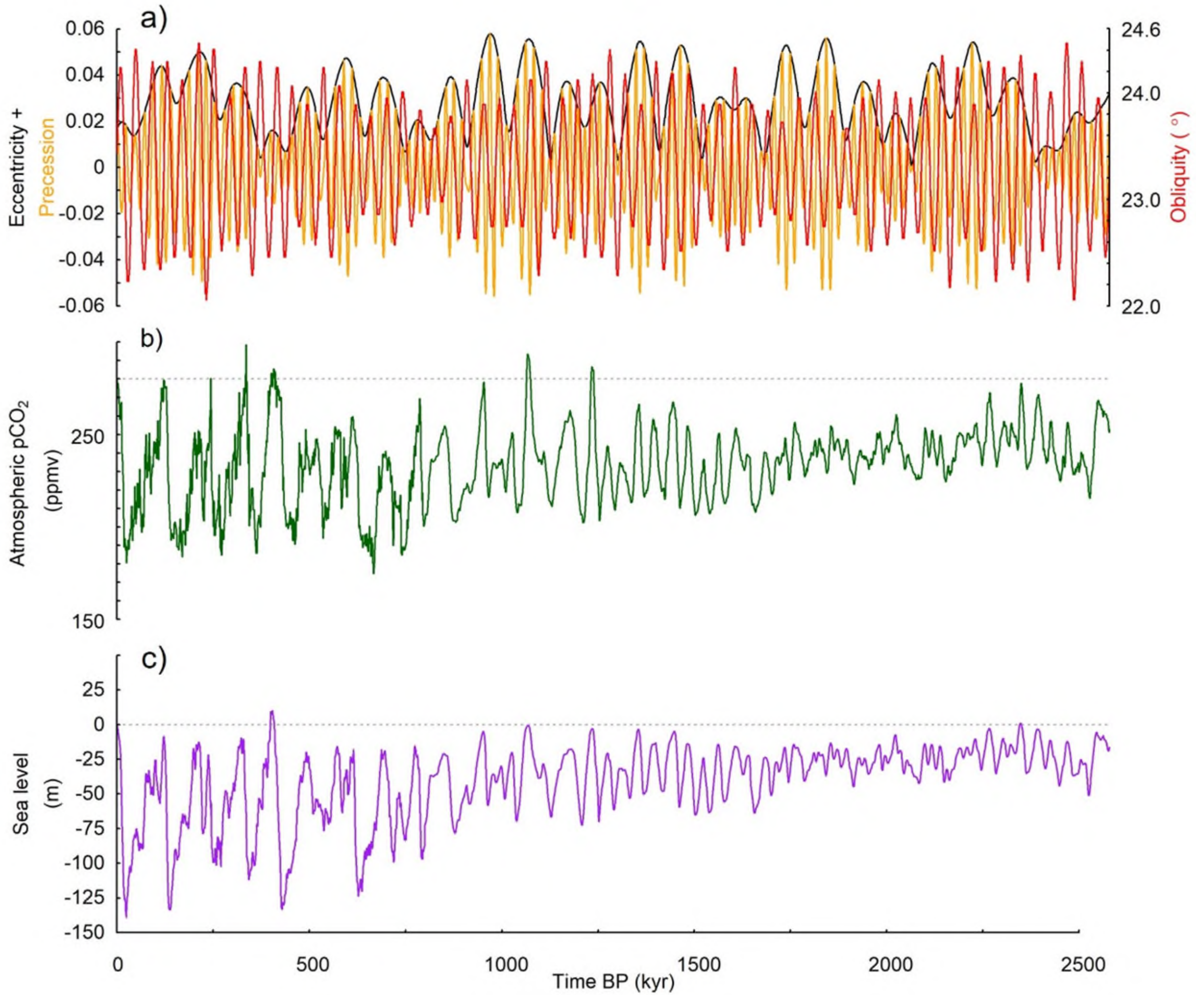

Figure S8: Forcing data. Climate forcing data used as input to the emulator for the last 2.58 million years (Myr): a) Orbital variations[24], showing eccentricity and precession on the left axis, and obliquity on the right axis; b) Atmospheric carbon dioxide (CO_2) concentrations, constructed from composite records from Antarctic ice cores[15] over the last 800 thousand years (kyr) and based on a model-derived CO_2 signal for the remaining Pleistocene[25], where the preindustrial (PI) CO_2 is also shown (grey dotted line); c) Reconstructed global sea level, derived from ocean sediment core $\delta^{18}O$ data[26] over the last 800 kyr and model-derived sea level[27] for the remaining Quaternary, both of which are shown as an anomaly compared with PI (grey dotted line).

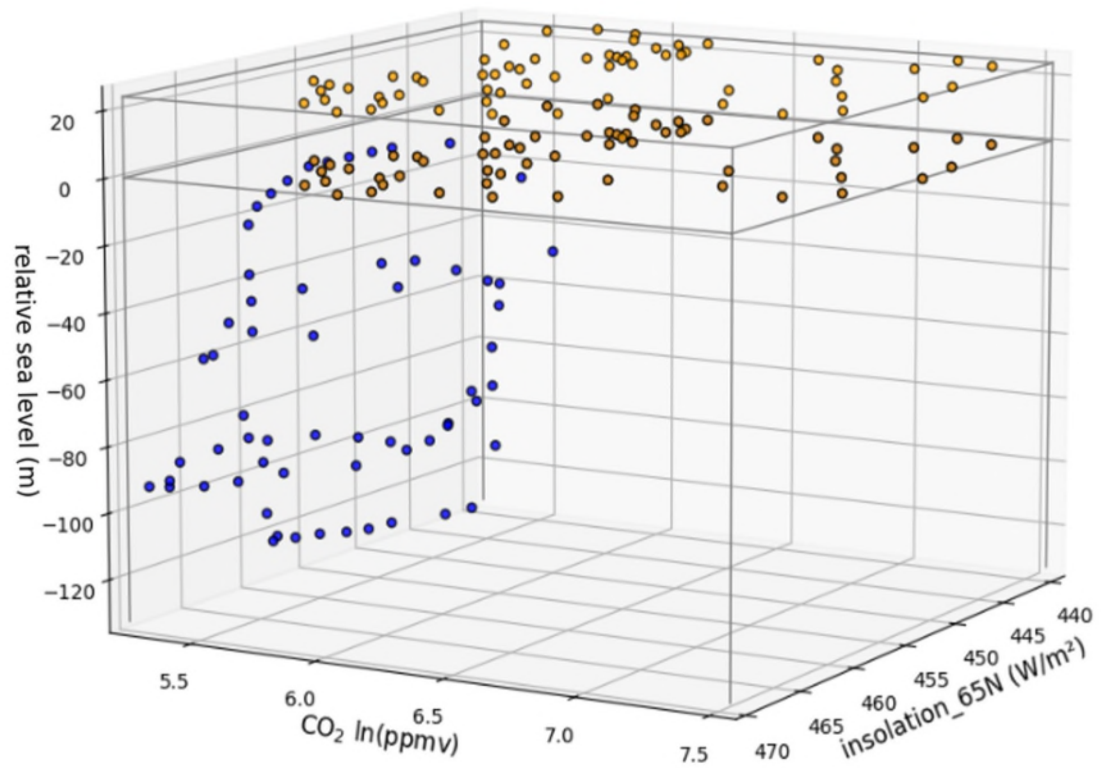

Fig. S9: Training simulations. HadCM3 training simulations going into the interglacial and glacial emulator. Each dot shows the HadCM3 training simulations according to relative sea level, log atmospheric carbon dioxide (CO_2) and insolation at 65°N, categorised according to whether relative sea level is at (red), above (yellow), or below (blue) 0 m. The interglacial emulator is trained on the climate model simulations at the red and orange dots, and the glacial emulator is trained on the simulations at the red and blue dots.

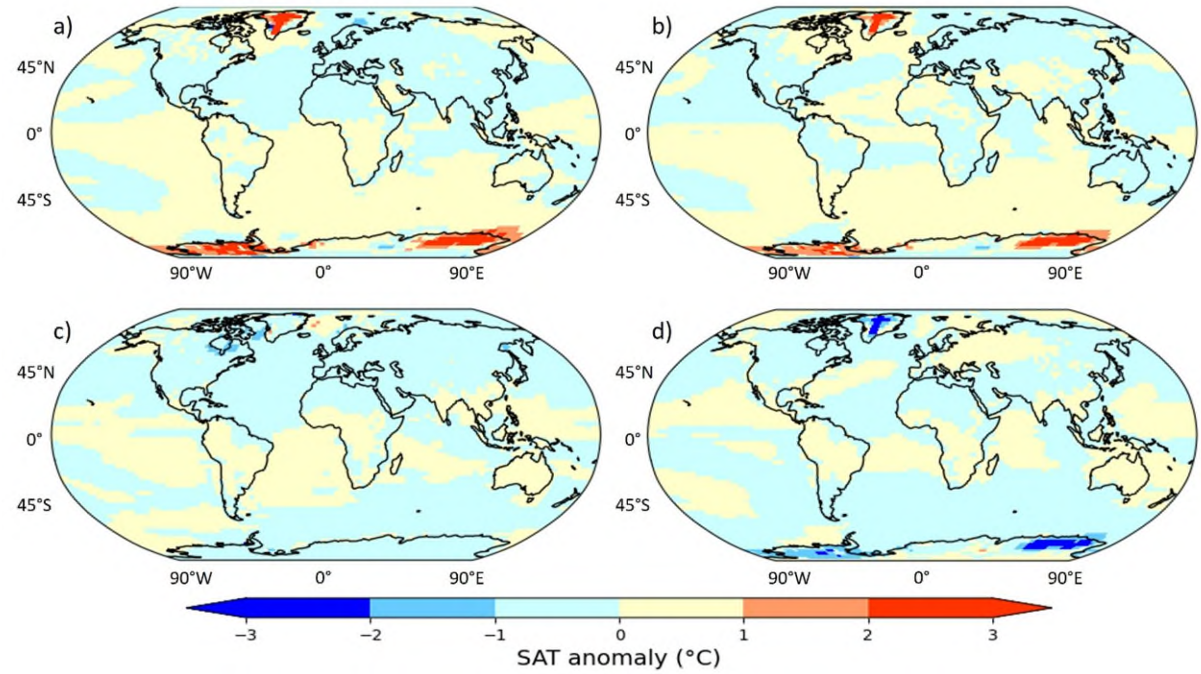

Fig. S10: Combined versus separate emulator. Surface air temperature (SAT anomalies (compared to the preindustrial control (PI, i.e. 0 thousand years (kyr)), °C) for two climate states, and using two approaches to running the emulator: a) climate state with global sea level (GSL) = +5 m, separate emulator approach; b) climate state with GSL = +5 m, combined emulator approach; c) climate state with GSL = -5 m, separate emulator approach; d) climate state with GSL = -5 m, combined emulator approach.

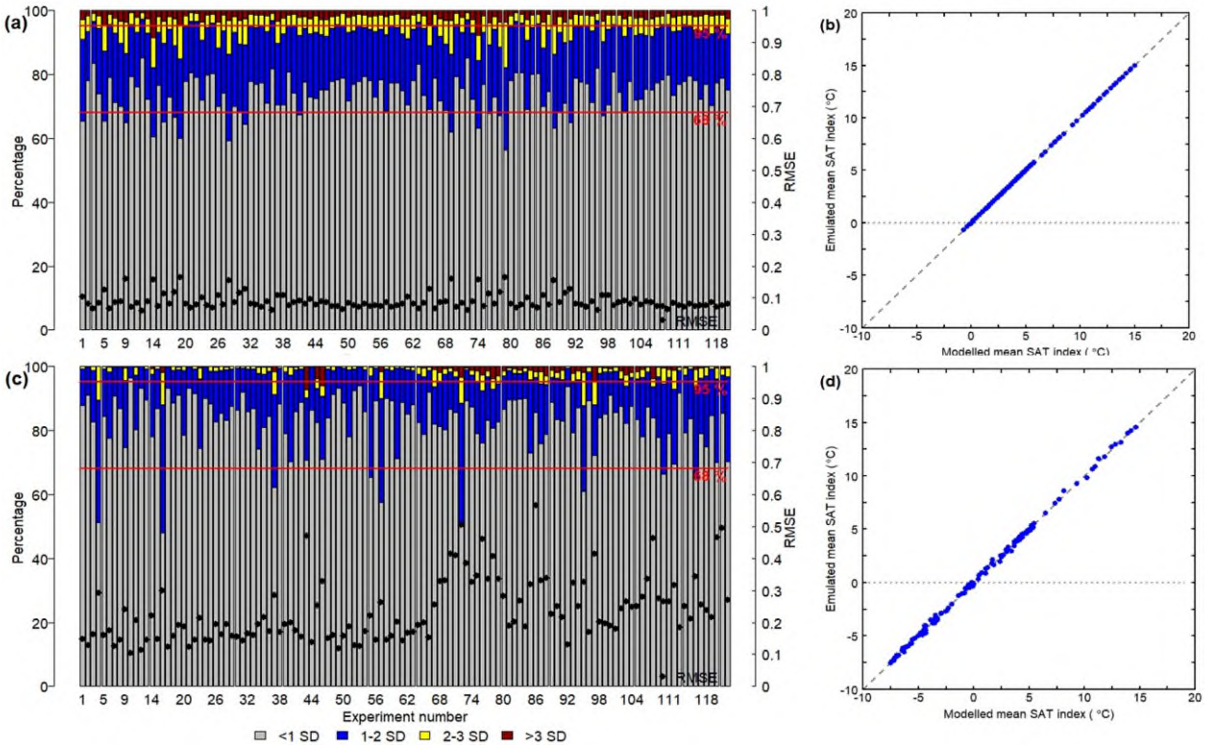

Figure S11: Emulator evaluation. Emulator performance for the interglacial emulator (top panel) and glacial emulator (bottom panel), both calibrated on surface air temperature (SAT) data, and HadCM3 training simulations according to relative sea level (bottom panel). In (a) and (c), bars give the percentage of grid boxes for which the emulator predicts the SAT of the left-out experiment to within 1, 2, 3 and more than 3 SD. Also shown is the root mean squared error (RMSE) for the experiments (black circles). Red lines indicate 68 and 95%. In (b) and (d), mean annual SAT index (°C) is calculated by the emulator and the climate model. The 1:1 line (dashed) is included for reference. Note: this is the mean value for the climate model output data grid assuming all grid boxes are of equal size, hence not taking into account variations in grid box area. SAT is shown as an anomaly compared with the pre-industrial (PI) control simulation.

REFERENCES

1. Tierney, J. E., Zhu, J., King, J., Malevich, S. B., Hakim, G. J. & Poulsen, C. J. (2020). Glacial cooling and climate sensitivity revisited. Nature, 584, 569–573, https://doi.org/10.1038/s41586-020-2617-x
2. Osman, M. B., Tierney, J. E., Zhu, J., Tardif, R., Hakim, G. J., King, J. & Poulsen, C. J. (2021). Globally resolved surface temperatures since the Last Glacial Maximum. Nature, 599, 239–244. https://doi.org/10.1038/s41586-021-03984-4
3. Lunt, D. J., Chandan, D., Haywood, A. M., Lunt, G. M., Rougier, J. C., Salzmann, U., Schmidt, G. A. & Valdes, P. J. (2021). Multi-variate factorisation of numerical simulations. Geoscientific Model Development. 14, 4307-4317. https://doi.org/10.5194/gmd-14-4307-2021
4. Haywood, A. M., Dowsett, H. J., Dolan, A. M., Rowley, D., Abe-Ouchi, A., Otto-Bliesner, B., Chandler, M. A., Hunter, S. J., Lunt, D. J., Pound, M. & Salzmann, U. (2016). The Pliocene Model Intercomparison Project (PlioMIP) Phase 2: scientific objectives and experimental design. Climate of the Past, 12(3), 663-675. doi:10.5194/cp-12-663-2016
5. Stone, E. J., Lunt, D. J., Rutt, I. C. & Hanna, E. (2010). Investigating the sensitivity of numerical model simulations of the modern state of the Greenland ice-sheet and its future response to climate change. Cryosphere, 4(3), 397-417. doi:10.5194/tc-4-397-2010
6. Winkelmann, R., Levermann, A., Ridgwell, A. & Caldeira, K. (2015). Combustion of available fossil fuel resources sufficient to eliminate the Antarctic Ice Sheet. Science Advances, 1(8). doi:10.1126/sciadv.1500589
7. Schmidt, P., Lund, B., Naslund, J. O., & Fastook, J. (2014). Comparing a thermo-mechanical Weichselian Ice Sheet reconstruction to reconstructions based on the sea level equation: aspects of ice configurations and glacial isostatic adjustment. Solid Earth, 5(1), 371-388. doi:10.5194/se-5-371-2014
8. Tarasov, L., Lecavalier, B. S., Hank, K., and Pollard, D. (2025), The glacial systems model (GSM) Version 25G, Geosci. Model Dev., 18, 9565–9603, https://doi.org/10.5194/gmd-18-9565-2025
9. Abe-Ouchi, A., Saito, F., Kageyama, M., Braconnot, P., Harrison, S. P., Lambeck, K., Otto-Bliesner, B. L., Peltier, W. R., Tarasov, L., Peterschmitt, J.-Y. & Takahashi, K. (2015). Ice-sheet configuration in the CMIP5/PMIP3 Last Glacial Maximum

experiments. Geoscientific Model Development, 8(11), 3621-3637. doi:10.5194/gmd-8-3621-2015

10. Colleoni, F., Wekerle, C., Naslund, J. O., Brandefelt, J. & Masina, S. (2016). Constraint on the penultimate glacial maximum Northern Hemisphere ice topography (approximate to 140 kyrs BP). Quaternary Science Reviews, 137, 97-112. doi:10.1016/j.quascirev.2016.01.024
11. Cox, P. M., Betts, R. A., Jones, C. D., Spall, S. A., & Totterdell, I. J. (2002). Modelling vegetation and the carbon cycle as interactive elements of the climate system. In R. Pearce (Ed.), Meteorology at the Millennium (pp. 259-279). San Diego CA, USA: Academic Press.
12. IPCC (2021). Climate Change 2021: The Physical Science Basis. Contribution of Working Group I to the Sixth Assessment Report of the Intergovernmental Panel on Climate Change[Masson-Delmotte, V., P. Zhai, A. Pirani, S.L. Connors, C. Péan, S. Berger, N. Caud, Y. Chen, L. Goldfarb, M.I. Gomis, M. Huang, K. Leitzell, E. Lonnoy, J.B.R. Matthews, T.K. Maycock, T. Waterfield, O. Yelekçi, R. Yu, and B. Zhou (eds.)]. Cambridge University Press, Cambridge, United Kingdom and New York, NY, USA.
13. Trauth, M. H., Asrat, A., Fischer, M. L., Hopcroft, P. O., Foerster, V., Kaboth-Bahr, S., Kindermann, K., Lamb, H. F., Marwan, N., Maslin, M. A., Schaebitz, F. & Valdes, P. J. (2024). Early warning signals of the termination of the African Humid Period(s). Nat Commun., 15, 3697, https://doi.org/10.1038/s41467-024-47921-1
14. Sun Y., Yin, Q., Crucifix, M., Clemens, S. C., Araya-Melo, P., Liu, W., Qiang, X., Liu, Q., Zhao, H., Liang, L., Chen, H., Li, Y., Zhang, L., Dong, G., Li, M., Zhou, W., Berger, A. & An, Z. (2019), Diverse manifestations of the mid-Pleistocene climate transition, Nature Communications, (10), doi:10.1038/s41467-018-08257-9
15. Bereiter, B., Eggleston, S., Schmitt, J., Nehrbass-Ahles, C., Stocker, T. F., Fischer, H., Kipfstuhl, S. & Chappellaz, J. (2015). Revision of the EPICA Dome C CO_2 record from 800 to 600 kyr before present. Geophysical Research Letters, 42(2), 542-549. doi:10.1002/2014gl061957
16. Jouzel, J., Masson-Delmotte, V., Cattani, O., Dreyfus, G., Falourd, S., Hoffmann, G., . . . Wolff, E. W. (2007). Orbital and millennial Antarctic climate variability over the past 800,000 years. Science, 317 (5839), 793-796. doi:10.1126/science.1141038

17. Wang, Y. J., Cheng, H., Edwards, R. L., An, Z. S., Wu, J. Y., Shen, C. C., & Dorale, J. A. (2001). A high-resolution absolute-dated Late Pleistocene monsoon record from Hulu Cave, China. Science, 294(5550), 2345-2348. doi:10.1126/science.1064618
18. Wang, Y. J., Cheng, H., Edwards, R. L., Kong, X. G., Shao, X. H., Chen, S. T., Wu, J., Jiang, X., Wang, X. & An, Z. (2008). Millennial- and orbital-scale changes in the East Asian monsoon over the past 224,000 years. Nature, 451(7182), 1090-1093. doi:10.1038/nature06692
19. Cheng, H., Edwards, R. L., Sinha, A., Spotl, C., Yi, L., Chen, S. T., Kelly, M., Kathayat, G., Wang, X., Li, X., Kong, X., Wang, Y., Ning, Y. & Zhang, H. (2016). The Asian monsoon over the past 640,000 years and ice age terminations. Nature, 534(7609), 640-+. doi:10.1038/nature18591
20. Herbert, T. D., Schuffert, J. D., Andreasen, D., Heusser, L., Lyle, M., Mix, A., Ravelo, A. C., Stott, L. D. & Herguera, J. C. (2001). Collapse of the California Current during glacial maxima linked to climate change on land. Science. 293 (5527):71-6. doi: 10.1126/science.1059209
21. Dyez, K. A., Ravelo, A. C. & A. C. (2016). Evaluating drivers of Pleistocene eastern tropical Pacific sea surface temperature. Paleoceanography and Paleoclimatology. https://doi.org/10.1002/2015PA002873
22. Wara, M. W., Ravelo, A. C & Delaney, M. L. (2005). Permanent El Niño-Like Conditions During the Pliocene Warm Period. Science. 309 (5735): 758-761. DOI: 10.1126/science.1112596
23. Hayward, B. W., Scott, G. H., Crundwell, M. P., Kennett, J. P., Carter, L., Neil, H. L., Sabaa, A. T., Wilson, K., Rodger, J. S., Schaefer, G., Grenfell, H. R. & Li, Q. (2008). The effect of submerged plateaux on Pleistocene gyral circulation and sea-surface temperatures in the Southwest Pacific. Global and Planetary Change. 63 (4): 309-316. https://doi.org/10.1016/j.gloplacha.2008.07.003
24. Laskar, J., Robutel, P., Joutel, F., Gastineau, M., Correia, A. C. M., & Levrard, B. (2004). A long-term numerical solution for the insolation quantities of the Earth. Astronomy & Astrophysics, 428(1), 261-285. doi:10.1051/0004-6361:20041335
25. Van de Wal, R. S. W., de Boer, B., Lourens, L. J., Köhler, P. & Bintanja, R. (2011). Reconstruction of a continuous high-resolution CO2 record over the past 20 million years. Climate of the Past, 7, 1459-1469, https://doi.org/10.5194/cp-7-1459-2011
26. Spratt, R. M., & Lisiecki, L. E. (2016). A Late Pleistocene sea level stack. Climate of the Past, 12(4), 1079-1092. doi:10.5194/cp-12-1079-2016

27. de Boer, B., van de Wal, R. S. W., Bintanja, R., Lourens, L. J., & Tuenter, E. (2010). Cenozoic global ice-volume and temperature simulations with 1-D ice-sheet models forced by benthic $\delta^{18}O$ records. Annals of Glaciology, 51, 23-33, https://doi.org/10.3189/172756410791392736
